# Associations of night shift work with weight gain among female nurses in The Netherlands: results of a prospective cohort study

**DOI:** 10.5271/sjweh.4185

**Published:** 2024-10-01

**Authors:** Henriëtte M van Duijne, Nina E Berentzen, Roel CH Vermeulen, Jelle J Vlaanderen, Hans Kromhout, Katarzyna Jóźwiak, Anouk Pijpe, Matti A Rookus, Flora E van Leeuwen, Michael Schaapveld

**Affiliations:** 1 Department of Epidemiology, Netherlands Cancer Institute, Amsterdam, The Netherlands.; 2 Institute for Risk Assessment Sciences, Utrecht University, Utrecht, The Netherlands.; 3 Institute of Biostatistics and Registry Research, Brandenburg Medical School Theodor Fontane, Germany; 4 Association of Dutch Burn Centers, Red Cross Hospital, Beverwijk, The Netherlands.; 5 Amsterdam University Medical Centre, Location VU University Medical Centre, Department of Plastic, Reconstructive, and Hand Surgery, Amsterdam Movement Sciences, Amsterdam, The Netherlands.

**Keywords:** BMI, body mass index, circadian rhythm, menopausal status, nursing, obesity, occupational health, occupational environment, overweight

## Abstract

**Objectives:**

This study aimed to prospectively investigate associations of working night shifts with weight gain in the Nightingale Study, a large cohort of female nurses.

**Methods:**

This study included 36 273 registered nurses, who completed questionnaires in 2011 and 2017. Cumulative number of nights, mean number of nights/month and consecutive number of nights/month in 2007–2011 were assessed. We used Poisson regression to estimate multivariable-adjusted incidence rate ratios (IRR) of >5% weight gain from 2011 to 2017 among all participants and assess risk of development of overweight/obesity (BMI≥25 kg/m^2^) among women with healthy baseline body mass index. The reference group consisted of women who never worked nights.

**Results:**

Overall, working night shifts in 2007–2011 was associated with >5% weight gain [IRR 1.07, 95% confidence interval (CI) 1.01–1.13]. Associations differed by menopausal status in 2011, with an increased risk of gaining >5% weight limited to postmenopausal women who worked nights (IRR 1.23, 95% CI 1.10–1.38). Postmenopausal women had an increased risk of >5% weight gain when they worked on average ≥4 nights/month (4–5: IRR 1.29, 95% CI 1.09–1.52, ≥6: IRR 1.27, 95% CI 1.11–1.47) or ≥4 consecutive nights/month (IRR 1.37, 95% CI 1.19–1.58), compared to postmenopausal women who never worked nights. For postmenopausal women with healthy weight at baseline, night shift work was associated with an increased risk of overweight/obesity at follow-up (IRR 1.24, 95% CI 1.03–1.50).

**Conclusions:**

Working night shifts was associated with a slightly increased risk of weight gain and overweight/obesity development among women who were postmenopausal at study inclusion. Our findings emphasize the importance of health promotion to maintain a healthy weight among (postmenopausal) night workers.

In Europe, 19% of the employed population works night shifts (1), a proportion that has been stable since 2005 (2). Working during the night may increase the risk of cardiovascular disease (3, 4), metabolic syndrome (5) and diabetes (6–9). Weight gain due to working at night may play a role in these associations (10), and obesity has been associated with increased risk of various types of cancer (11). Working night shifts may cause weight gain through impaired glucose metabolism as a consequence of circadian disruption (12–14). However, studies examining the association between (night) shift work and weight change have shown inconsistent results. A systematic review and meta-analysis of 26 observational studies found that shift work was associated with a risk of overweight [relative risk (RR) 1.25, 95% confidence interval (CI) 1.08–1.44] and obesity (RR 1.17, 95% CI 1.12–1.22) (15). This is in line with a systematic review of 17 longitudinal studies performed between 1996 and 2012 (16). However, no associations were found in two other systematic reviews, although the included studies partly overlapped with those included in the earlier reviews (10, 17). Another meta-analysis did not observe a difference in obesity risk between shift workers and non-shift workers [odds ratio (OR) 1.05, 95% CI 0.97–1.14], nor when the analysis was restricted to females (18). However, when separating by world regions, increased risks for obesity were observed in Europe, the USA and Australia. A recent systematic review and meta-analysis of 63 studies among males and females in multiple professions, found increased body mass index (BMI) for shift workers [standardized mean difference (SMD) 0.10 kg/m^2^, 95% CI 0.07–0.13], compared to day workers (19). However, for females, no difference in BMI was found when comparing those working shifts to day workers. Differences in study design and populations, outcome definition (weight gain versus overweight or obesity) and definition of (night) shift work may explain these conflicting findings. Most studies assessed either the association of current night shift work versus day work exposure or the change from day work to night work exposure and risk of weight gain/obesity. The heterogeneity between studies demonstrates a need for prospective studies with detailed exposure assessment (including duration, frequency and intensity of (night) shift work). The aim of the current study was to prospectively assess the association of night shift work using multiple exposure metrics with subsequent weight gain and development of overweight or obesity in a cohort of female nurses.

## Methods

The Nightingale Study is a prospective cohort study initiated in The Netherlands among 59 947 female registered nurses aged 18–65 years at inclusion (20). At baseline (2011), between 6 October 2011 and 1 February 2012, the women completed a web-based questionnaire including a detailed assessment of work exposures and risk factors for chronic diseases such as lifestyle and reproductive factors. Of these, 62.5% (N=37 470) completed a follow-up questionnaire in 2017. In this questionnaire, the questions were generally the same but considered the exposure period 2012–2017. In the current study, we included participants who completed both the baseline and follow-up questionnaire. Compared to non-responders to the follow-up questionnaire, the women included in the current study were on average somewhat older at baseline [median age 49.9, interquartile range (IQR) 41.8–55.9, years versus 46.8, IQR 36.1–54.1, years)] and the mean BMI of these women was slightly lower [24.8, standard deviation (SD) 4.0, kg/m^2^ versus 25.1, SD 4.3, kg/m^2^]. Frequencies of overweight and obesity differed only slightly between responders and non-responders, with 29.5% and 10.0% being overweight or obese among responders to the follow-up questionnaire versus 30.1% and 12.3% among non-responders, respectively. All participates who had missing data for either night shift work (ever/never: N=15) or weight change (N=807) were excluded. In addition, we excluded women who were pregnant at baseline (N=375), as weight before pregnancy was not assessed. The final study sample for the current analysis included 36 273 women. The Institutional Review Board of The Netherlands Cancer Institute approved the Nightingale Study (Nightingale Studie, 2011, codes PTC10.2692 and PTC11.1972/JA), and all participants provided written informed consent.

### Assessment of night shift work

Night shift work was assessed in the first questionnaire (2011) through questions on job history, including each (historical) job, period-specific night work exposure (e.g. per calendar year). We did not include potential night shift work exposure during the educational period. A woman was considered to have ever conducted night work when she had worked at least one night shift (≥1 hour 24:00–05:00 hours) per month for at least six months. As we hypothesized the strongest association would be seen between recent night work and short-term weight gain, we defined the night shift work exposure period at the time of filling in the baseline questionnaire into recent exposure (having worked night shifts in the period 2007–2011) and current exposure (night shift work exposure in 2011). The main exposure metrics, for each of the two periods, were night work status, average number of nights worked per month, average number of consecutive nights worked per month and cumulative number of night shifts worked during the exposure period. Women who had worked night shifts, but not in the exposure period of interest (2007–2011 and 2011) were defined as former night shift workers. We based the categorization of continuous night shift work variables on tertiles among all participants who worked during nights. We performed a two-stage multiple imputation by chained equations (MICE) procedure in which, first, all missing values in covariates other than the night shift work exposures variables were imputed (stage 1) resulting in 40 imputed datasets. Subsequently missing values on the night shift work variables were imputed in participants who had performed night shifts 5 times in each of the 40 imputed datasets (stage 2), resulting in 200 imputed datasets [40 (stage 1) × 5 (stage2)] (21). Approximately 33% of the participants who indicated having ever worked night shifts did not complete (part of) the detailed period-specific night shift work section, resulting in missing values for cumulative night shift work metrics. However, for all night shift workers, we were able to derive years of employment in which they worked night shifts from the occupational history section, which we used as a predictor in the imputation procedure. Parameter estimates from the 200 imputed datasets were pooled according to a modified Rubin’s rules (22, 23). Further description of the two-stage MICE procedure is provided in the supplementary material, URL, Methods 1.

### Weight gain and development of overweight/obesity

Height (cm) and weight (kg) were reported in 2011 and 2017. We calculated BMI as body weight divided by squared height (kg/m^2^), which was calculated for all participants using baseline height. BMI was categorized as underweight (<18.5 kg/m^2^), healthy weight (18.5–24.9 kg/m^2^), overweight (25.0–29.9 kg/m^2^) and obesity (≥30.0 kg/m^2^) (24). We assessed the proportion of moderate (>5%) weight gain in all participants. We chose >5% weight gain as the measure for clinically relevant weight gain as 5% weight gain is associated with increased risks of cardiovascular disease and metabolic syndrome (25), and it has been used in other epidemiological studies assessing weight loss, breast cancer, hip arthroplasty and health behaviors (26–29). Also, we assessed the development of overweight/obesity among participants with healthy weight at baseline (N=21 566). We compared women who developed overweight/obesity, based on the BMI categories described above with women who maintained a healthy weight from 2011 to 2017.

### Statistical analysis

Continuous variables were presented as means (SD) or median (IQR) for normally and non-normally distributed variables, respectively, and categorical variables were presented as proportions. Weight gain of >5% during follow-up and development of overweight or obesity during follow-up were analyzed according to night shift work (categories) using a Poisson regression model with robust error variance to estimate RR with incidence rate ratio (IRR) (30). Poisson regression instead of logistic regression was used as the odds ratio (OR) may overestimate the risk ratio when outcomes are not rare (ie, incidence >10%). A trend over categories of night shift work was estimated for the night shift workers only, by assigning exposure categories the median value within that category of exposure and subsequently including this variable as a continuous variable in the model. For all analyses, we present age- and multivariable-adjusted models, which included, besides age, all variables that changed the regression coefficient for the association between (at least one of the categories of) cumulative number night shifts between 2007 and 2011 and the outcome of >5% weight gain by >10%, as rule of thumb of confounding, compared to the age-adjusted model. The potential confounders, assessed at baseline, were menopausal status (pre- or postmenopausal), sports activity (0, 1–2, ≥3 hours per week), alcohol consumption (0, 10–40, >40–60, >60–100, >100 grams per week), smoking status (current, former, never), marital status (married, living as married or living together; divorced or living separated; widow; living apart together; single; living at parents), highest education achieved (intermediate vocational education/community college, higher vocational/professional education/college/university of applied science, and university or higher), chronotype (defined as diurnal preference or an individual’s preference for timing daily activities in a given 24-hour day (31), assessed by asking how a respondent would describe herself and categorized into: definitely a morning person, more a morning than an evening person, more an evening than a morning person, definitely an evening person, no specific type), number of children (0–10), sleep duration (<6, 6–9, >9 hours) (32), current job load (sitting, standing/walking, heavy), average monthly household income of the participant’s residential address postal code at baseline (continuous) and night work during the follow-up period 2011–2017 (yes/no). To evaluate potential effect modification, we added interaction terms to the statistical models of night shift work variables with the potential effect modifier. We did this for age at baseline (18–35; 36–45; 46–55; 56–65 years), menopausal status at baseline (premenopausal; postmenopausal), menopausal status at follow-up for women who were premenopausal at baseline, chronotype at baseline (no specific type, early, more early than late, more late than early, late), employment status at baseline (no work, work, retired) and night work during the follow-up period 2011–2017 (yes/no). Besides the main analysis, which was based on multiply imputed data, we also performed a complete case analysis in which all participants with missing values in any of the variables of interest and confounders were omitted. A two-sided P-value of <0.05 was considered statistically significant. All analyses were conducted using STATA Statistical Software Release 15 (StataCorp, 2009, College Station, Texas).

## Results

At baseline, 6060 women had never performed night shift work and 10 021 women had performed night work in the five years preceding 2011, of whom 7668 (77%) women still worked night shifts in 2011. Among women who never worked nights, 24.5% gained >5% weight during the follow-up period. For women who worked night shifts between 2007 and 2011, this was 29.5%. Among women with healthy weight at baseline, 14.0% of the never night workers developed overweight/obesity compared to 16.5% among women who worked night shifts between 2007 and 2011 (table 1). Women who worked night shifts during 2007–2011 were more often definite evening types than never night workers (15.5% compared to 7.8%, respectively) and more often indicated their job load as mostly sitting (31.0% compared to 26.0%, respectively) (supplementary table S1). There were no appreciable differences in other characteristics between the groups.

**Table 1:** Characteristics at baseline (2011) and follow-up (2017) by night work status. [SD=standard deviation; IQR= interquartile range].

		Study sample (N=36 273) Median age 49.9 (IQR 41.8–56.0) years			Never night workers (N=6060) Median age 50.1 (IQR 0.6-56.8) years			Night workers <2007 (N=10 856) Median age 52.1 (IQR 26.1–57.3) years			Night workers 2007–2011 (N=10 021) Median age 45.0 (IQR 34.1–52.1) years	
		N (%)	Mean (SD)		N (%)	Mean (SD)		N (%)	Mean (SD)		N (%)	Mean (SD)
Menopausal status												
	Premenopausal	18 622 (51.3)			3133 (51.7)			4532 (41.7)			6606 (65.9)	
	Postmenopausal	12 432 (34.3)			2158 (35.6)			4525 (41.7)			2104 (21.0)	
	Missing	5219 (14.4)			769 (12.7)			1799 (16.6)			1311 (13.1)	
Body mass index at 18 years (kg/m^2^)			21.2 (2.8)			21.1 (2.8)			21.1 (2.7)			21.4 (2.9)
Weight (kg)			71.3 (12.2)			70.5 (11.8)			71.2 (12.2)			71.6 (12.5)
Height (cm)			169.6 (6.3)			169.7 (6.4)			169.4 (6.2)			170.0 (6.3)
Body mass index (kg/m^2^)			24.8 (4.0)			24.5 (3.8)			24.9 (4.0)			24.8 (4.1)
	<18.5	374 (1.0)			85 (1.4)			83 (0.8)			113 (1.1)	
	18.5–24.9	21 566 (59.5)			3756 (62.0)			6321 (58.2)			6038 (60.3)	
	25–29.9	10 686 (29.5)			1679 (27.7)			3349 (30.8)			2808 (28.0)	
	≥30	3647 (10.1)			540 (8.9)			1103 (10.2)			1062 (10.6)	
Characteristics after 5.5 years follow-up (2017)												
Weight (kg)			72.4 (12.9)			71.6 (12.5)			72.3 (12.7)			73.1 (13.3)
Body mass index (kg/m^2^)			25.2 (4.2)			24.9 (4.1)			25.2 (4.2)			25.3 (4.4)
	<18.5	406 (1.1)			85 (1.4)			103 (1.0)			108 (1.1)	
	18.5–24.9	19 813 (54.6)			3474 (57.3)			5 870 (54.1)			5451 (54.4)	
	25–29.9	11 530 (31.8)			1836 (30.3)			3 546 (32.7)			3129 (31.2)	
	≥30	4524 (12.5)			665 (11.0)			1337 (12.3)			1333 (13.3)	
Change during follow-up (2011–2017)												
>5% weight gain		9478 (26.1)			1485 (24.5)			2632 (24.2)			2961 (29.5)	
Weight change during follow-up (kg)			1.2 (5.7)			1.1 (5.5)			0.9 (5.4)			1.5 (6.0)
Weight gain during follow-up (kg)			4.6 (4.2)			4.5 (4.2)			4.3 (3.8)			4.9 (4.4)
From healthy (18.5–24.9 kg/m^2^) to overweight/obese (≥25 kg/m^2^)		3219 (14.9)			527 (14.0)			892 (14.1)			998 (16.5)	

The IRR of gaining >5% weight during follow-up was slightly higher among women who worked night shifts between 2007 and 2011 than women who never worked night shifts [multivariable-adjusted IRR (IRR_adj_) 1.07, 95% CI 1.01–1.13] (table 2). This association between night shift work exposure and >5% weight gain was significantly modified (P=0.040) by menopausal status in 2011, but not by age, chronotype, employment status, menopausal status in 2017 or night work during follow-up. Therefore, the analyses were stratified according to menopausal status in 2011. For premenopausal women, there was no difference in IRR of gaining >5% weight during follow-up when comparing women who worked nights between 2007 and 2011 to women who never worked nights (IRR_adj_ 1.02, 95% CI 0.96–1.08; table 2). IRR was also not significant for any of the cumulative night work metrics. For women who were postmenopausal in 2011, a slightly increased rate of gaining >5% was found for both women who had performed night shift work <2007 and night shift work in 2007–2011 (night shift work <2007: IRR_adj_ 1.18, 95% CI 1.06–1.30 and night shift work in 2007–2011: IRR_adj_ 1.23, 95% CI 1.10–1.38). We observed increased risk of >5% weight gain for women who worked ≥4 nights per month on average (4–5 nights: IRR_adj_ 1.29, 95% CI 1.09–1.52 and ≥6 nights: IRR_adj_ 1.27, 95% CI 1.11–1.47). For both cumulative number of nights and mean number of consecutive nights per month, no trends were observed for increasing exposure and risk of weight gain (cumulative: 1–121 nights IRR_adj_ 1.24, 95% CI 1.05–1.47 and ≥241 nights IRR_adj_ 1.30, 95% CI 1.13–1.49; consecutive: 1–2 nights IRR_adj_ 1.20, 95% CI 1.01–1.41 and ≥4 nights IRR_adj_ 1.37, 95% CI 1.19–1.58).

**Table 2:** Associations of night shift work exposure 2007–2011 with moderate weight gain (>5%) during 5.5 years follow-up, for the entire study population and menopausal status subgroups, based on multiply-imputed data (N=36 273). [IRR=incidence rate ratio; CI= confidence interval; ref= reference category; no.=number].

Night work exposure in 2007–2011		All participants			Premenopausal in 2011			Postmenopausal in 2011	
		IRR (95% CI) ^a^	IRR (95% CI) ^b^		IRR (95% CI) ^a^	IRR (95% CI) ^c^		IRR (95% CI) ^a^	IRR (95% CI) ^c^
Night work status									
	Never	1 [ref.]	1 [ref.]		1 [ref.]	1 [ref.]		1 [ref.]	1 [ref.]
	Night work <2007	1.08 (1.02–1.13)	1.07 (1.02–1.13)		1.03 (0.97–1.09)	1.03 (0.97–1.10)		1.18 (1.07–1.30)	1.18 (1.06–1.30)
	Night work in 2007–2011	1.11 (1.05–1.17)	1.07 (1.01–1.13)		1.04 (0.98–1.11)	1.02 (0.96–1.08)		1.32 (1.18–1.48)	1.23 (1.10–1.38)
Comparison within night workers									
	Night work <2007	1 [ref.]	1 [ref.]		1 [ref.]	1 [ref.]		1 [ref.]	1 [ref.]
	Night work in 2007–2011	1.03 (0.98–1.07)	1.00 (0.95–1.04)		1.01 (0.96–1.07)	0.99 (0.94–1.04)		1.12 (1.03–1.22)	1.05 (0.96–1.14)
Mean no. nights per month (tertiles)		1.02 (1.01–1.03)	1.01 (1.00–1.02)		1.01 (1.01–1.02)	1.01 (0.99–1.02)		1.03 (1.02–1.04)	1.02 (1.01–1.03)
	Never	1 [ref.]	1 [ref.]		1 [ref.]	1 [ref.]		1 [ref.]	1 [ref.]
	1–3	1.10 (1.04–1.17)	1.06 (0.99–1.13)		1.04 (0.98–1.12)	1.03 (0.96–1.10)		1.21 (1.02–1.42)	1.15 (0.97–1.36)
	4–5	1.09 (1.02–1.16)	1.05 (0.98–1.12)		1.02 (0.94–1.09)	1.00 (0.92–1.07)		1.38 (1.17–1.63)	1.29 (1.09–1.52)
	≥6	1.20 (1.12–1.29)	1.12 (1.04–1.20)		1.12 (1.04–1.22)	1.06 (0.98–1.15)		1.40 (1.22–1.61)	1.27 (1.11–1.47)
	*P for trend*	*0.030*	*0.173*		*0.060*	*0.326*		*0.138*	*0.322*
Cumulative no. nights (tertiles)		1.00 (1.00–1.00)	1.00 (1.00–1.00)		1.00 (1.00–1.00)	1.00 (0.99–1.00)		1.00 (0.99–1.00)	1.00 (1.00–1.00)
	Never	1 [ref.]	1 [ref.]		1 [ref.]	1 [ref.]		1 [ref.]	1 [ref.]
	1–121	1.09 (1.02–1.17)	1.05 (0.98–1.13)		1.02 (0.95–1.10)	1.01 (0.94–1.09)		1.30 (1.10–1.54)	1.24 (1.05–1.47)
	122–240	1.09 (1.02–1.16)	1.05 (0.98–1.12)		1.04 (0.97–1.11)	1.02 (0.95–1.09)		1.20 (1.02–1.42)	1.14 (0.97–1.35)
	≥241	1.20 (1.12–1.29)	1.12 (1.04–1.21)		1.12 (1.03–1.21)	1.06 (0.98–1.15)		1.44 (1.26–1.65)	1.30 (1.13–1.49)
	*P for trend*	*0.018*	*0.148*		*0.040*	*0.225*		*0.104*	*0.337*
Mean no. consecutive nights per month (tertiles)		1.03 (1.02–1.05)	1.02 (1.01–1.03)		1.02 (1.01–1.04)	1.01 (0.99–1.03)		1.06 (1.04–1.09)	1.05 (1.02–1.07)
	Never	1 [ref.]	1 [ref.]		1 [ref.]	1 [ref.]		1 [ref.]	1 [ref.]
	1–2	1.11 (1.03–1.19)	1.06 (0.99–1.14)		1.04 (0.96–1.12)	1.02 (0.95–1.11)		1.26 (1.06–1.48)	1.20 (1.01–1.41)
	3	1.09 (1.01–1.16)	1.04 (0.96–1.11)		1.03 (0.96–1.11)	1.00 (0.93–1.08)		1.21 (1.03–1.42)	1.12 (0.95–1.32)
	≥4	1.17 (1.10–1.25)	1.11 (1.04–1.19)		1.09 (1.01–1.17)	1.04 (0.97–1.12)		1.50 (1.31–1.72)	1.37 (1.19–1.58)
	*P for trend*	*0.063*	*0.165*		*0.168*	*0.459*		*0.038*	*0.960*

^a^ Adjusted for age at baseline. ^b^ Adjusted for age, smoking status, menopausal status, alcohol consumption, sports activity, highest education achieved, chronotype, average monthly household income, sleep duration, marital status, number of children and job load at baseline. ^c^ Adjusted for age, smoking status, alcohol consumption, sports activity, highest education achieved, chronotype, average monthly household income, sleep duration, marital status, number of children and job load at baseline.

**Figure 1:**
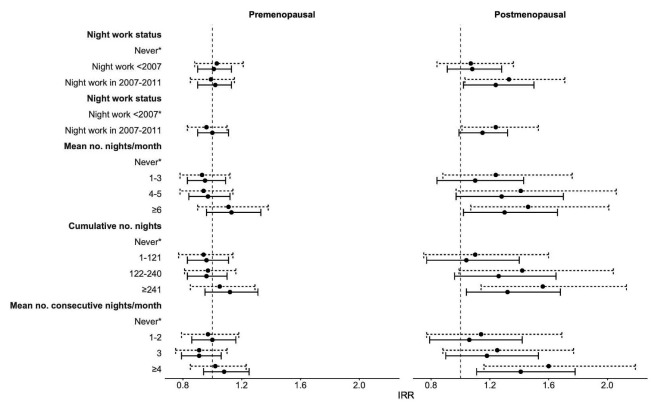
Forest plot showing the associations of night shift work exposure 2007–2011 with development of overweight or obesity during 5.5 year follow-up in a subgroup of nurses with healthy weight at baseline, by menopausal status at baseline. Adjusted for age, smoking status, alcohol consumption, sports activity, highest education achieved, chronotype, average monthly household income, sleep duration, marital status, number of children and job load at baseline. Note: *reference category. Incidence rate ratios (IRR) estimates and 95% confidence intervals (CI) based on multiply-imputed data are indicated by solid black lines (N=21 566); IRR estimates 95% CI based on observed data are indicated by dashed black lines (N=12 787).

Among women with a healthy BMI at baseline, there was no significant difference in the rate of developing overweight/obesity during follow-up for women who worked at night in the period 2007–2011 compared to women who never worked night shifts (IRR_adj_ 1.06, 95% CI 0.96–1.18) (supplementary table S2). Although there was no statistically significant difference for premenopausal women, the risk of developing overweight/obesity was higher for postmenopausal women who worked nights between 2007 and 2011 compared with women who never worked nights (premenopausal women: IRR_adj_ 1.01, 95% CI 0.90–1.13 and postmenopausal women: IRR_adj_ 1.24, 95% CI 1.03–1.50) (figure 1 and supplementary table S2). Among premenopausal women, none of the exposure metrics for night shift work showed an association with development of overweight/obesity. However, postmenopausal women who worked >241 nights between 2007 and 2011 (IRR_adj_ 1.32, 95% CI 1.04–1.68), on average ≥6 nights per month (IRR_adj_ 1.30, 95% CI 1.02–1.66) or ≥4 consecutive nights per month (IRR_adj_ 1.41, 95% CI 1.12–1.79) had an increased risk to develop overweight/obesity compared with women who never worked nights. Although the risk estimates for developing overweight/obesity tended to increase with higher level of exposure to night shift work, only the highest exposure categories showed statistically significantly increased risks and the tests for trends over categories of exposure did not reach statistical significance. IRR rates were similar for night work exposure in 2011 compared to night work exposure in 2007–2011 in both the analyses of risk of >5% weight gain and development of overweight/obesity (supplementary tables S3 and S4. The complete case analyses differed slightly with regard to IRR point estimates and resulted in wider CI, but the directions of the associations remained unchanged (supplementary tables S5 and S6 for night work exposure between 2007 and 2011 and supplementary tables S7 and S8 for night work exposure in 2011).

## Discussion

In this prospective study of female nurses, women who were postmenopausal in 2011 and performed night shift work experienced a slightly increased risk of weight gain during 5.5 years of follow-up compared to postmenopausal women who did never work during nights. Among premenopausal women, there was no association of night shift work exposure with weight gain or with development of overweight/obesity. Among postmenopausal women, the risk of gaining weight increased with higher night work exposure, as did the risk of development of overweight/obesity among participants who had a healthy weight at baseline. We emphasize that the effect sizes for the association of night shift work exposure with weight gain among postmenopausal women were small. Most risk estimates attenuated and the majority of trends disappeared after confounder adjustment, highlighting the importance of careful multivariable adjustment.

Comparison of our results with other studies is complicated by differences in exposure assessment, as our study focused on detailed night work exposure assessment and not on changes in work schedule (eg, from day to night shifts) as other studies did. However, our findings are in line with results of a recent prospective study in which male and female nurses who only worked night shifts showed a larger increase in BMI compared to nurses who only worked during days (33). Another study showed no association between night shift work and weight gain among male and female employees in the Dutch working population; however follow-up was only one year in this study (34). A study among female nurses and midwives adjusted for menopausal status supported the relationship between night shift work and weight gain (35). None of these studies assessed potential effect modification by menopausal status of the association between night shift work and weight gain.

Sleep deprivation could be a possible mediator in the association between night shift work and weight gain or the development of overweight or obesity among postmenopausal women. In general, night shift workers often experience lower sleep quality, lack of sleep, and broken night's sleep (36). In addition, postmenopausal women frequently suffer from sleep abnormalities, due to the influence of multiple factors such as menopausal hormonal changes (37, 38). Insufficient sleep may lead to an increase in appetite and consequently weight gain (39, 40). While a meta-analysis showed that energy intake does not differ between day and shift workers (41), it has been suggested that decreased metabolic efficiency resulting from sleep deprivation (42, 43) may also play a role in weight gain among night workers (28, 44). Since the metabolic rate may also be reduced after menopause (45), it is plausible that especially postmenopausal women experience weight gain in response to night shift work. Future studies could investigate sleep duration, sleep quality and sleep medication use (46) as potential mediators in the association between night shift work and weight gain among postmenopausal women.

The finding that night shift work was associated with increased weight gain among post- but not premenopausal women is novel and should be confirmed by other studies. There is some evidence that shift work tolerance (the ability to adapt to shift work without adverse consequences) decreases after the age of 40–45, possibly related to changes in circadian rhythms or physical fitness (47–49). Menopause is characterized by endocrine and physiologic changes, including changes in circadian rhythm regularity (50). For example, the nocturnal secretion of the pineal hormone melatonin sharply declines during middle age (51, 52). Night work-related disruption of the endogenous circadian rhythm may add to the dysrhythmia of menopause, thereby potentially further increasing the likelihood of metabolic disturbances (10). In addition, the role of mediating lifestyle behaviors such as reduced sleep and physical activity and changed eating habits should be clarified in future studies.

When interpreting our results, the strengths and limitations of our study must be considered. In our current study, potential changes in night work and other factors (ie, lifestyle) after 2011 may have affected weight gain during follow-up. Although we found no evidence of confounding and effect modification by night work status (yes/no) during the follow-up period, misclassification of cumulative night work variables cannot be excluded. Women who were premenopausal at baseline may have become postmenopausal during follow-up. However, we found no evidence for effect modification by menopausal status in 2017 for women who were premenopausal at baseline. Also, by testing for effect modification by night shift work status at follow-up, we ruled out that especially postmenopausal women stopped working nights during the follow-up period. Our data are self-reported and are based on questionnaires as is common in studies on shift work and health. A previous study showed valid results of self-reported data on night shift work exposure compared to objective data (53). Although differential exposure misclassification related to weight seems unlikely, we cannot exclude the possibility of non-differential misclassification of self-reported night shift work and weight. Another limitation is the substantial number of women for whom it is unknown whether they worked night shifts between 2007 and 2011 (9336 of 30 213 ever night workers). However, all these women did work nights during their working life. The generalizability of our results may be limited to women with a similar occupational background. Although most studies focused on night shift work, disruption of circadian rhythm may also be caused by morning, evening, and sleep shifts instead of night shifts alone. Depending on an individual’s chronotype, these shift types may also collide with the individual’s biological night. A recent systematic review and meta-analysis found higher BMI among shift workers in several occupations (working outside work hours: 06:00–18:00 hours, Monday to Friday) compared to day workers (19). Therefore, it would be of interest for future studies to consider all types of shifts as well as to quantify individual circadian disruption resulting from these shifts. Our study does have some important strengths. We could use a large cohort with prospective assessment of weight change. Furthermore, no previous study has been able to investigate such a large extent of different exposure metrics of night shift work in relation to prospective weight gain. Our results therefore may have a substantive contribution to the body of knowledge in this field. Other strengths include detailed information on potential confounders, such as chronotype, physical activity, marital status, average monthly household income and smoking status (10, 18).

In conclusion, we observed slightly increased risks of weight gain as well as development of overweight or obesity for postmenopausal women who performed night shift work. A higher mean number of nights, a higher cumulative number of nights worked, and a higher number of consecutive nights worked per month were all associated with higher risk of weight gain among postmenopausal women. As overweight and obesity are risk factors for several chronic diseases, our findings highlight the importance of promoting healthy weight among night shift workers in the occupational environment.

## Supplementary material

Supplementary material
