# Supplementary material for: Associations of night shift work with weight gain among female nurses in The Netherlands: results of a prospective cohort study
Source: Scand J Work Environ Health. 2024 Sep 30;50(7):536–44. doi: 10.5271/sjweh.4185 (PMC11473151; doi:10.5271/sjweh.4185)
Supplement: Supplementary material [file SJWEH-50-536-S001.pdf]

Associations of night shift work with weight gain among female nurses in The Netherlands: results of a prospective cohort study[1]

by Henriëtte M van Duijne, MSc, Nina E Berentzen, PhD, Roel CH Vermeulen, PhD, Jelle J Vlaanderen, PhD, Hans Kromhout, PhD, Katarzyna Jóźwiak, PhD, Anouk Pijpe, PhD, Matti A Rookus, PhD, Flora E van Leeuwen, PhD, Michael Schaapveld, PhD [1]

1. Supplemental material
2. Correspondence to: Michael Schaapveld, PhD, Department of Epidemiology, Netherlands Cancer Institute, Plesmanlaan 121, 1066 CX Amsterdam, The Netherlands. [E-mail: m.schaapveld@nki.nl].

Supplementary Methods 1: Two-stage Multiple imputation

Table S1. Baseline (2011) characteristics of the study sample and by night work status (2007-2011)

Table S2. Associations of night shift work exposure between 2007 and 2011 with development of overweight or obesity during 5.5 year follow-up in a subgroup of nurses with healthy weight at baseline, for the entire study population and for menopausal status subgroups , based on multiply-imputed data (n=21 566)

Table S3. Associations of night shift work exposure in 2011 with moderate weight gain (>5%) during 5.5 year follow-up by menopausal status at baseline, based on multiply-imputed data (n=36 273)

Table S4. Associations of night shift work exposure in 2011 with development of overweight or obesity during 5.5 year follow-up in a subgroup of nurses with healthy weight at baseline, by menopausal status at baseline, based on multiply-imputed data (n=21 566)

Table S5. Associations of night shift work exposure between 2007 and 2011 with moderate weight gain (>5%) during 5.5 year follow-up, for the entire study population and for menopausal status subgroups, based on observed data (n= 16 040)

Table S6. Associations of night shift work exposure between 2007 and 2011 with development of overweight or obesity during 5.5 year follow-up in a subgroup of nurses with healthy weight at baseline, for the entire study population and for menopausal status subgroups, based on observed data (n=9652)

Table S7. Associations of night shift work exposure in 2011 with moderate weight gain (>5%) during 5.5 year follow-up by menopausal status at baseline, based on observed data (n=16 040)

Table S8. Associations of night shift work exposure in 2011 with development of overweight or obesity during 5.5 year follow-up in a subgroup of nurses with healthy weight at baseline, by menopausal status at baseline, based on observed data (n=9652)

Supplementary Methods 1: Two-stage Multiple imputation

In the present study, 26% (15 396/36 273) of the eligible participants had at least one missing value for the set of variables considered relevant for the subsequent Poisson regression analyses. The fractions of missing values in individual variables were low to moderate (0%–26%). We used two-stage multiple imputation by chained equations (MICE) to replace missing values and generate multiple complete datasets (22). The first set of variables multiply imputed (i.e. stage I) was a set of covariate variables for the entire study population. The imputation model in stage I included all variables that were a priori selected for the subsequent analysis models (listed below) and potential predictors of the incomplete variables ("auxiliary" variables). In this stage, 40 data sets were created using chained equations. The second set of variables multiply imputed (i.e. stage II) contained night work variables for night workers only. The imputation model in stage II included predictors of the incomplete nigh work variables ("auxiliary" variables), (i.e. work-related factors). In stage II, 5 data sets were created using each imputed dataset in stage I, resulting in a total of 40 times 5 (or 200) imputed data sets. In both stages, variables with the least number of missing values were imputed first. We used the following statistical models for multiple imputation, depending on variable type and distribution (nominal, ordinal, interval, ratio or count).

Stage 1: imputation of variables for all participants

MICE was conducted to impute missing values of covariates for all 36 273 women.

- Linear regression for a continuous variable was used to impute physical activity level, sleep duration and average monthly household income. Because of its highly skewed distribution, physical activity level and average monthly household income were transformed using a logarithm transformation and transformed back to the original scale after imputation. Subsequently, physical activity level was categorized (no sports activity, 1-2 hours per week, ≥3 hours per week) and sleep duration was categorized as <6, 6-9, >9 hours per night).
- Predictive mean matching for a continuous variable with 5 nearest neighbors was used to impute lifetime work duration and lifetime work duration as nurse.
- Negative binomial regression for a count variable was used to impute gram of alcohol drunk per week in the past year (alcohol abstainers, 10-40 gram per week, >40-60 gram per week, >60-100 gram per week, >100 gram per week).
- Logistic regression for a binary variable was used to impute menopausal status (pre- or postmenopausal).

- Ordered logistic regression for an ordinal variable was used to impute smoking status (current, former, never), job load (sitting, standing/walking, heavy) and regularity in daily life (no, some, average, much regularity).
- Multinomial binomial regression for a nominal variable was used to impute chronotype (definitely a morning person, more a morning than an evening person, more an evening than a morning person, definitely an evening person, no specific type, I don't know) and marital status (married or living as married or living together, divorced or living separated, widow, living apart together, single, living at parents).
- Poisson regression was used to impute number of children (0-10).
- All imputation models were adjusted for age at baseline, height, weight at baseline, weight at follow-up, highest nursing degree (intermediate vocational education/community college, higher vocational/professional education/college/university of applied science, university or higher), and highest other education degree (intermediate vocational education/community college, higher vocational/professional education/college/university of applied science, university or higher).

Stage 2: imputation of variables for night workers only

MICE was conducted to impute missing values of exposure metrics for 9336 night workers.

- Predictive mean matching for a continuous variable with 5 nearest neighbors was used to impute number night work period records, time since night work quitting (years), mean number of nights worked per month between 2007 and 2011, mean number of consecutive nights worked per month between 2007 and 2011, cumulative number of nights worked between 2007 and 2011, mean number of nights worked per month in 2011 and mean number of consecutive nights worked per month in 2011.
- Logistic regression for a binary variable was used to impute status of night work in the past 6 months (no, yes), family as the reason for quitting night work (no, yes), irregular day-night rhythm as the reason for quitting night work (no, yes), older age as the reason for quitting night work (no, yes).
- Multinomial binomial regression for a nominal variable was used to impute switching to normal day-night rhythm (first sleep then switch back, switched back immediately, variable).
- Information on 5% weight gain and development of overweight/obesity were included in all imputation models as a dichotomous variable (5% weight gain occurred yes/no,

development overweight/obesity occurred yes/no) and all imputation models were adjusted for smoking status, alcohol consumption, sports activity, chronotype, average monthly household income, sleep duration, marital status, number of children and job load, which were imputed in stage I.

- Additionally, all imputation models were adjusted for age at baseline, highest nursing degree, highest other education degree, regularity in daily life, lifetime work duration and lifetime work duration as nurse- the last 3 variables were imputed in stage I.

Parameter estimates and their variance from analyzing the imputed datasets were pooled according to the modified Rubin's rules (22, 54, 55). Multiple imputations and subsequent Poisson regression analyses were performed in Stata version 15.1 (Stata Corp, College Station, TX, USA).

Table S1. Baseline (2011) characteristics of the study sample and by night work status (2007-2011)

	Study sample (n=36 273)	%	Mean (SD)	Never night workers (n=6060)	%	Mean (SD)	Night workers 2007-2011 (n=10 021)	%	Mean (SD)	Night workers <2007 (n=10 856)	%	Mean (SD)	Unknown whether night workers 2007-2011 (n=9336)	%	Mean (SD)
**Baseline characteristics (2011)**															
Education (highest achieved level)															
Intermediate vocational education community college	18 946	52.2%		3165	52.2%		5565	55.5%		5187	47.8%		5029	53.9%	
Higher vocational/professional education/ college/university of applied science	13 397	36.9%		2229	36.8%		3801	37.9%		40470	37.3%		3320	35.6%	
University or higher	3930	10.8%		666	11.0%		655	6.6%		1622	14.9%		987	10.6%	
Alcohol consumption															
Alcohol abstainers	16 473	45.4%		2724	45.0%		5210	52.0%		4365	40.2%		4174	44.7%	
10-40 gram per week	6788	18.7%		1089	18.0%		1945	19.4%		2060	19.0%		1694	18.1%	
>40-60 gram per week	4084	11.3%		685	11.3%		978	9.8%		1324	12.2%		1097	11.8%	
>60-100 gram per week	5134	14.2%		901	14.9%		1145	11.4%		1758	16.2%		1330	14.2%	
>100 gram per week	3668	10.1%		633	10.4%		720	7.2%		1310	12.1%		1004	10.8%	
Missing	127	0.4%		28	0.4%		23	0.2%		39	0.3%		37	0.4%	
Physical activity															
No participation in sport	2988	8.2%		490	8.1%		810	8.1%		843	7.8%		845	9.0%	
1-2 hours per week participation in sport	8850	24.4%		1572	25.9%		2612	26.1%		2,513	23.1%		2153	23.1%	
≥2 hours per week participation in sport	24 083	66.4%		3920	64.7%		6543	65.3%		7409	68.3%		6211	66.5%	
Missing	352	1.0%		78	1.3%		56	0.5%		91	0.8%		127	1.4%	
Smoking															
Never	17 424	48.0%		2974	49.1%		5460	54.5%		4726	43.5%		4264	45.7%	
Former	14 716	40.6%		2426	40.0%		3,357	33.5%		5008	46.1%		3925	42.0%	
Current	4074	11.2%		650	10.7%		1,191	11.9%		1106	10.2%		1127	12.1%	
Missing	59	0.2%		10	0.2%		13	0.1%		16	0.2%		20	0.2%	
Chronotype															
No preference	9128	25.2%		1520	25.1%		2594	25.9%		1584	24.5%		2360	25.3%	
Definite morning type	4756	13.1%		958	15.8%		886	8.8%		2799	14.6%		1328	14.2%	
Probable morning type	8534	23.5%		1536	25.4%		2094	20.9%		2654	25.8%		2105	22.6%	
Probable evening type	7834	21.6%		1181	19.5%		2464	24.6%		2201	20.3%		1988	21.3%	
Definite evening type	3850	10.6%		438	7.2%		1469	14.7%		1041	10.0%		902	9.7%	
Missing	2171	6.0%		427	7.1%		514	5.1%		577	5.3%		653	7.0%	
Job load															
No current job	5156	14.2%		1003	16.6%		479	4.8%		2146	19.8%		1528	16.4%	
Sitting	8471	23.4%		1650	27.2%		1081	10.8%		3634	33.5%		2106	22.6%	
Standing/walking	10 734	29.6%		1631	26.9%		4078	40.7%		2439	22.5%		2586	27.7%	
Heavy	8203	22.6%		1036	17.1%		3741	37.3%		1374	12.7%		2052	22.0%	

Missing	3709	10.2%		740	12.2%		642	6.4%		1263	11.6%		1064	11.4%	
Average monthly household income of the baseline postal code area , mean (SD)	32 151		2781.1 (856.6)	5368		2750.6 (875.0)	8835		2687.8 (735.7)	9660		2880.8 (920.7)	8288		2784.0 (870.5)
Sleep duration, mean (SD)	34 148		7.1 (0.9)	5631		7.2 (0.9)	9521		7.2 (0.9)	10 297		7.1 (0.9)	8699		7.1 (1.0)
<6 hours per night	1322	3.6%		202	3.3%		321	3.2%		432	4.0%		367	3.9%	
6-9 hours per night	32 587	89.8%		5384	88.8%		9130	91.1%		9809	90.4%		8264	88.5%	
>9 hours per night	239	0.7%		45	0.7%		70	0.7%		56	0.5%		68	0.7%	
Missing	2125	5.9%		429	7.1%		500	5.0%		559	5.2%		637	6.8%	
Number of children, mean (SD)	35 044		1.8 (1.3)	5842		1.8 (1.3)	9697		1.6 (1.3)	10 504		1.9 (1.2)	9001		1.8 (1.3)

All variables are presented as *N* (%) or mean (SD) or median [IQR]. Abbreviations: SD, standard deviation- n, number- IQR, interquartile range.

Table S2. Associations of night shift work exposure between 2007 and 2011 with development of overweight or obesity during 5.5 year follow-up in a subgroup of nurses with healthy weight at baseline, for the entire study population and for menopausal status subgroups , based on multiply-imputed data (n=21 566)

	All participants		Premenopausal in 2011		Postmenopausal in 2011	
	IRR (95% CI)[a]	IRR (95% CI)[b]	IRR (95% CI)[a]	IRR (95% CI)[c]	IRR (95% CI)[a]	IRR (95% CI)[c]
**Night work exposure in 2007-2011**						
Night work status						
Never	1 [ref.]	1 [ref.]	1 [ref.]	1 [ref.]	1 [ref.]	1 [ref.]
Night work <2007	1.04 (0.95- 1.14)	1.03 (0.94-1.13)	1.00 (0.89- 1.13)	1.01 (0.90- 1.13)	1.09 (0.92- 1.28)	1.08 (0.92- 1.28)
Night work in 2007-2011	1.14 (1.04- 1.26)	1.06 (0.96-1.18)	1.06 (0.95- 1.19)	1.01 (0.90- 1.13)	1.40 (1.16- 1.68)	1.24 (1.03- 1.50)
Comparison within night workers						
Night work <2007	1 [ref.]	1 [ref.]	1 [ref.]	1 [ref.]	1 [ref.]	1 [ref.]
Night work in 2007-2011	1.10 (1.01- 1.19)	1.03 (0.95-1.13)	1.06 (0.96- 1.17)	1.00 (0.90- 1.11)	1.29 (1.12- 1.48)	1.14 (0.99- 1.32)
Mean no. nights per month (tertiles)	1.03 (1.02-1.04)	1.02 (1.00-1.03)	1.02 (1.01-1.04)	1.01 (0.99-1.03)	1.04 (1.02-1.06)	1.03 (1.00-1.05)
Never	1 [ref.]	1 [ref.]	1 [ref.]	1 [ref.]	1 [ref.]	1 [ref.]
1-3	1.10 (0.98- 1.24)	0.99 (0.87-1.12)	1.02 (0.89- 1.17)	0.95 (0.83- 1.10)	1.24 (0.96- 1.61)	1.10 (0.84- 1.44)
4-5	1.12 (0.99- 1.27)	1.03 (0.90-1.18)	1.03 (0.89- 1.18)	0.97 (0.84- 1.13)	1.44 (1.09- 1.90)	1.28 (0.97- 1.70)
≥6	1.33 (1.17- 1.52)	1.18 (1.03-1.35)	1.24 (1.06- 1.45)	1.13 (0.96- 1.33)	1.53 (1.21- 1.95)	1.30 (1.02- 1.66)
*P*trend	0.012	0.028	0.016	0.043	0.169	0.288
Cumulative no. nights (tertiles)	1.00 (1.00-1.00)	1.00 (1.00-1.00)	1.00 (1.00-1.00)	1.00 (0.99-1.00)	1.00 (1.00-1.00)	1.00 (1.00-1.00)
Never	1 [ref.]	1 [ref.]	1 [ref.]	1 [ref.]	1 [ref.]	1 [ref.]
1-121	1.07 (0.94- 1.22)	0.99 (0.87-1.13)	1.02 (0.88- 1.18)	0.96 (0.83- 1.12)	1.15 (0.86- 1.54)	1.04 (0.77- 1.40)
122-240	1.12 (0.99- 1.26)	1.02 (0.90-1.16)	1.02 (0.89- 1.17)	0.96 (0.84- 1.10)	1.41 (1.08- 1.84)	1.26 (0.96- 1.66)
≥241	1.33 (1.17- 1.51)	1.18 (1.03-1.35)	1.22 (1.05- 1.43)	1.12 (0.96- 1.31)	1.56 (1.24- 1.97)	1.32 (1.04- 1.68)
*P*trend	0.007	0.027	0.015	0.045	0.073	0.184
Mean no. consecutive nights per month (tertiles)	1.05 (1.02-1.07)	1.03 (1.00-1.06)	1.03 (0.99-1.06)	1.02 (0.99-1.05)	1.09 (1.05-1.14)	1.06 (1.01-1.11)
Never	1 [ref.]	1 [ref.]	1 [ref.]	1 [ref.]	1 [ref.]	1 [ref.]
1-2	1.14 (1.00- 1.30)	1.03 (0.90-1.18)	1.07 (0.93- 1.24)	1.00 (0.86- 1.17)	1.19 (0.89- 1.58)	1.06 (0.79- 1.42)
3	1.08 (0.95- 1.23)	0.98 (0.86-1.11)	0.98 (0.85- 1.13)	0.92 (0.79- 1.06)	1.36 (1.06- 1.76)	1.18 (0.90- 1.53)
≥4	1.28 (1.13- 1.44)	1.16 (1.02-1.32)	1.16 (1.01- 1.33)	1.08 (0.94- 1.25)	1.63 (1.29- 2.05)	1.41 (1.12- 1.79)
*P*trend	0.065	0.068	0.211	0.254	0.046	0.070

Abbreviations: IRR: incidence rate ratio- CI: confidence interval- ref: reference category- no: number.

[a]adjusted for age at baseline

[b]adjusted for age, smoking status, menopausal status, alcohol consumption, sports activity, highest education achieved, chronotype, average monthly household income, sleep duration, marital status, number of children and job load at baseline

[c]adjusted for age, smoking status, alcohol consumption, sports activity, highest education achieved, chronotype, average monthly household income, sleep duration, marital status, number of children and job load at baseline

Table S3. Associations of night shift work exposure in 2011 with moderate weight gain (>5%) during 5.5 year follow-up by menopausal status at baseline, based on multiply-imputed data (n=36 273)

	Premenopausal in 2011		Postmenopausal in 2011	
	IRR (95% CI)[a]	IRR (95% CI)[b]	IRR (95% CI)[a]	IRR (95% CI)[b]
**Night work exposure in 2011**				
Night work status				
Never	1 [ref.]	1 [ref.]	1 [ref.]	1 [ref.]
Night work <2011	1.04 (0.98-1.10)	1.03 (0.98-1.10)	1.19 (1.08-1.31)	1.18 (1.07-1.30)
Night work in 2011	1.04 (0.98-1.10)	1.01 (0.95-1.08)	1.34 (1.19-1.51)	1.23 (1.09-1.39)
Comparison within night workers				
Night work <2011	1 [ref.]	1 [ref.]	1 [ref.]	1 [ref.]
Night work in 2011	1.00 (0.96-1.05)	0.98 (0.93-1.03)	1.13 (1.03-1.24)	1.05 (0.95-1.15)
Mean no. nights per month (tertiles)	1.01 (1.00-1.02)	1.00 (0.99-1.02)	1.03 (1.02-1.05)	1.02 (1.01-1.04)
Never	1 [ref.]	1 [ref.]	1 [ref.]	1 [ref.]
1-3	1.04 (0.97-1,12)	1.02 (0.94-1.10)	1.17 (0.97-1.40)	1.09 (0.90-1.33)
4-5	1.02 (0.95-1.11)	1.00 (0.92-1.08)	1.42 (1.18-1.69)	1.30 (1.08-1.56)
≥6	1.12 1.02-1.22)	1.05 (0.95-1.15)	1.47 (1.27-1.70)	1.30 (1.12-1.51)
*P*trend	0.056	0.333	0.043	0.157
Mean no. consecutive nights per month (tertiles)	1.02 (1.01-1.04)	1.01 (0.99-1.03)	1.07 (1.04-1.10)	1.05 (1.02-1.08)
Never	1 [ref.]	1 [ref.]	1 [ref.]	1 [ref.]
1-2	1.01 (0.94-1.10)	1.00 (0.92-1.08)	1.25 (1.04-1.50)	1.17 (0.98-1.41)
3	1.03 (0.95-1.11)	0.99 (0.91-1.08)	1.24 (1.03-1.48)	1.12 (0.93-1.35)
≥4	1.11 (1.02-1.19)	1.05 (0.97-1.14)	1.54 (1.32-1.79)	1.37 (1.17-1.59)
*P*trend	0.035	0.149	0.039	0.121

Abbreviations: IRR: incidence rate ratio- CI: confidence interval- ref: reference category- no: number.

[a]adjusted for age at baseline

[b]adjusted for age, smoking status, alcohol consumption, sports activity, highest education achieved, chronotype, average monthly household income, sleep duration, marital status, number of children and job load at baseline

Table S4. Associations of night shift work exposure in 2011 with development of overweight or obesity during 5.5 year follow-up in a subgroup of nurses with healthy weight at baseline, by menopausal status at baseline, based on multiply-imputed data (n=21 566)

	Premenopausal in 2011		Postmenopausal in 2011	
	IRR (95% CI)[a]	IRR (95% CI)[b]	IRR (95% CI)[a]	IRR (95% CI)[b]
**Night work exposure in 2011**				
Night work status				
Never	1 [ref.]	1 [ref.]	1 [ref.]	1 [ref.]
Night work <2011	1.00 (0.90-1.12)	1.00 (0.90-1.12)	1.10 (0.93-1.29)	1.09 (0.92-1.28)
Night work in 2011	1.07 (0.95-1.20)	1.02 (0.90-1.15)	1.44 (1.19-1.76)	1.27 (1.04-1.55)
Comparison within night workers				
Night work <2011	1 [ref.]	1 [ref.]	1 [ref.]	1 [ref.]
Night work in 2011	1.07 (0.97-1.18)	1.01 (0.91-1.12)	1.32 (1.13-1.53)	1.16 (0.99-1.36)
Mean no. nights per month (tertiles)	1.02 (1.01-1.04)	1.01 (0.99-1.03)	1.05 (1.03-1.08)	1.03 (1.01-1.06)
Never	1 [ref.]	1 [ref.]	1 [ref.]	1 [ref.]
1-3	1.03 (0.89-1.18)	0.96 (0.83-1.11)	1.16 (0.86-1.57)	1.03 (0.75-1.40)
4-5	1.06 (0.91-1.23)	1.00 (0.85-1.17)	1.55 (1.14-2.10)	1.35 (0.99-1.84)
≥6	1.24 (1.05-1.48)	1.13 (0.94-1.35)	1.67 (1.30-2.14)	1.39 (1.07-1.80)
*P*trend	0.021	0.049	0.037	0.088
Mean no. consecutive nights per month (tertiles)	1.03 (1.00-1.07)	1.02 (0.98-1.05)	1.11 (1.06-1.16)	1.07 (1.01-1.12)
Never	1 [ref.]	1 [ref.]	1 [ref.]	1 [ref.]
1-2	1.06 (0.92-1.24)	1.00 (0.85-1.17)	1.28 (0.95-1.73)	1.15 (0.84-1.56)
3	1.00 (0.85-1.16)	0.92 (0.78-1.08)	1.36 (1.02-1.81)	1.15 (0.85-1.55)
≥4	1.19 (1.03-1.38)	1.10 (0.94-1.29)	1.72 (1.34-2.21)	1.45 (1.12-1.87)
*P*trend	0.144	0.182	0.092	0.178

Abbreviations: IRR: incidence rate ratio- CI: confidence interval- ref: reference category- no: number.

[a]adjusted for age at baseline

[b]adjusted for age, smoking status, alcohol consumption, sports activity, highest education achieved, chronotype, average monthly household income, sleep duration, marital status, number of children and job load at baseline

Table S5. Associations of night shift work exposure between 2007 and 2011 with moderate weight gain (>5%) during 5.5 year follow-up, for the entire study population and for menopausal status subgroups, based on observed data (n=16 040)

	All participants			Premenopausal in 2011			Postmenopausal in 2011		
	n >5% weight gain during follow-up/n total	IRR (95% CI)[a]	IRR (95% CI)[b]	n >5% weight gain during follow-up/n total	IRR (95% CI)[a]	IRR (95% CI)[c]	n >5% weight gain during follow-up/n total	IRR (95% CI)[a]	IRR (95% CI)[c]
**Night work exposure in 2007-2011**									
Night work status									
Never	877/3495	1 [ref.]	1 [ref.]	642/2093	1 [ref.]	1 [ref.]	235/1402	1 [ref.]	1 [ref.]
Night work <2007	1515/6313	1.07 (0.99-1.15)	1.07 (0.99-1.14)	940/3209	1.05 (0.97-1.15)	1.06 (0.98-1.16)	575/3104	1.10 (0.96-1.26)	1.10 (0.96-1.26)
Night work in 2007-2011	1867/6232	1.08 (1.01-1.16)	1.04 (0.97-1.12)	1522/4699	1.02 (0.95-1.10)	1.00 (0.92-1.08)	345/1533	1.30 (1.12-1.51)	1.20 (1.03-1.40)
Night worker, period unknown	1352/5335			879/2912			473/2423		
Comparison within night workers									
Night work <2007	1515/6313	1 [ref.]	1 [ref.]	940/3209	1 [ref.]	1 [ref.]	575/3104	1 [ref.]	1 [ref.]
Night work in 2007-2011	1867/6232	1.02 (0.95-1.08)	0.98 (0.92-1.04)	1522/4699	0.97 (0.90-1.04)	0.94 (0.87-1.01)	345/1533	1.19 (1.05-1.34)	1.09 (0.97-1.23)
Night worker, period unknown	1352/5335			879/2912			473/2423		
Mean no. nights per month (tertiles)		1.02 (1.01-1.03)	1.00 (0.99-1.00)		1.02 (1.00-1.03)	1.01 (0.99-1.02)		1.03 (1.01-1.04)	1.02 (0.99-1.03)
Never	877/3495	1 [ref.]	1 [ref.]	642/2093	1 [ref.]	1 [ref.]	235/1402	1 [ref.]	1 [ref.]
1-3	587/1953	1.07 (0.98-1.16)	1.05 (0.96-1.14)	609/1920	1.02 (0.93-1.11)	1.01 (0.92-1.11)	105/474	1.26 (1.02-1.55)	1.20 (0.98-1.48)
4-5	462/1513	1.01 (0.92-1.11)	0.99 (0.91-1.00)	509/1618	0.96 (0.87-1.05)	0.94 (0.85-1.04)	78/335	1.34 (1.07-1.68)	1.26 (1.01-1.58)
≥6	1456/5707	1.20 (1.10-1.32)	1.10 (1.00-1.21)	327/914	1.16 (1.04-1.29)	1.08 (0.97-1.20)	135/599	1.33 (1.10-1.60)	1.18 (0.98-1.43)
Missing	104/372			77/247			27/125		
Night worker, period unknown	1352/5335			879/2912			473/2423		
P-trend		0.028	0.175		0.011	0.101		0.756	0.867
Cumulative no. nights (tertiles)		1.00 (1.00-1.00)	1.00 (0.99-1.00)		1.00 (1.00-1.00)	1.00 (0.99-1.00)		1.00 (1.00-1.00)	1.00 (0.99-1.00)
Never	877/3495	1 [ref.]	1 [ref.]	642/2093	1 [ref.]	1 [ref.]	235/1402	1 [ref.]	1 [ref.]
1-121	569/1930	1.05 (0.96-1.14)	1.03 (0.94-1.12)	474/1502	0.99 (0.90-1.09)	0.98 (0.89-1.08)	95/428	1.28 (1.03-1.58)	1.23 (0.99-1.52)
122-240	702/2336	1.04 (0.95-1.13)	1.02 (0.93-1.11)	622/1940	1.00 (0.91-1.09)	0.99 (0.90-1.08)	80/396	1.17 (0.93-1.46)	1.11 (0.88-1.39)
≥241	492/1594	1.20 (1.09-1.32)	1.11 (1.01-1.22)	349/1010	1.12 (1.01-1.25)	1.05 (0.95-1.17)	143/584	1.43 (1.19-1.71)	1.26 (1.05-1.52)
Missing	104/372			77/247			27/125		
Night worker, period unknown	1352/5335			879/2912			473/2423		
P-trend		0.020	0.130		0.041	0.153		0.204	0.551
Mean no. consecutive nights per month (tertiles)		1.03 (1.01-1.05)	1.00 (0.99-1.00)		1.02 (0.99-1.04)	1.00 (0.99-1.03)		1.07 (1.03-1.10)	1.05 (1.01-1.08)
Never*	877/3495	1 [ref.]	1 [ref.]	642/2093	1 [ref.]	1 [ref.]	235/1402	1 [ref.]	1 [ref.]
1-2	496/1710	1.08 (0.98-1.18)	1.05 (0.96-1.16)	414/1326	1.02 (0.92-1.13)	1.02 (0.92-1.13)	82/384	1.22 (0.98-1.53)	1.19 (0.95-1.49)
3	568/1996	1.02 (0.93-1.12)	0.99 (0.90-1.08)	483/1538	0.99 (0.90-1.09)	0.97 (0.88-1.07)	85/458	1.07 (0.85-1.34)	0.98 (0.78-1.23)
≥4	689/2117	1.14 (1.05-1.25)	1.09 (0.99-1.18)	541/1569	1.05 (0.96-1.16)	1.01 (0.91-1.11)	148/548	1.58 (1.32-1.89)	1.42 (1.18-1.70)
Missing	114/409			84/266			30/143		
Night worker, period unknown	1352/5335			879/2912			473/2423		
P-trend		0.073	0.240		0.306	0.729		0.020	0.057

Abbreviations: IRR: incidence rate ratio- CI: confidence interval- ref: reference category- no: number.

[a]adjusted for age at baseline, [b]adjusted for age, smoking status, menopausal status, alcohol consumption, sports activity, highest education achieved, chronotype, average monthly household income, sleep duration, marital status, number of children and job load at baseline, [c]adjusted for age, smoking status, alcohol consumption, sports activity, highest education achieved, chronotype, average monthly household income, sleep duration, marital status, number of children and job load at baseline

Table S6. Associations of night shift work exposure between 2007 and 2011 with development of overweight or obesity during 5.5 year follow-up in a subgroup of nurses with healthy weight at baseline, for the entire study population and for menopausal status subgroups, based on observed data (n=9652)

	All participants			Premenopausal in 2011			Postmenopausal in 2011		
	n overweight or obesity follow-up/ n healthy weight at baseline	IRR (95% CI)[a]	IRR (95% CI)[b]	n overweight or obesity follow-up/ n healthy weight at baseline	IRR (95% CI)[a]	IRR (95% CI)[c]	n overweight or obesity follow-up/ n healthy weight at baseline	IRR (95% CI)[a]	IRR (95% CI)[c]
**Night work exposure in 2007-2011**									
Night work status									
Never	308/2187	1 [ref.]	1 [ref.]	222/1373	1 [ref.]	1 [ref.]	86/814	1 [ref.]	1 [ref.]
Night work <2007	519/3701	1.04 (0.91-1.18)	1.04 (0.91-1.18)	326/1991	1.02 (0.87-1.19)	1.03 (0.88-1.21)	193/1710	1.07 (0.84-1.36)	1.07 (0.84-1.36)
Night work in 2007-2011	620/3764	1.12 (0.99-1.27)	1.06 (0.93-1.21)	489/2948	1.02 (0.88-1.18)	0.99 (0.85-1.15)	131/816	1.52 (1.18-1.96)	1.33 (1.03-1.71)
Night worker, period unknown	490/3135			314/1779			176/1356		
Comparison within night workers									
Night work <2007	519/3701	1 [ref.]	1 [ref.]	326/1991	1 [ref.]	1 [ref.]	193/1710	1 [ref.]	1 [ref.]
Night work in 2007-2011	620/3764	1.08 (0.96-1.21)	1.02 (0.91-1.15)	489/2948	1.01 (0.88-1.15)	0.96 (0.83-1.10)	131/816	1.42 (1.16-1.74)	1.24 (1.01-1.53)
Night worker, period unknown	490/3135			314/1779			176/1356		
Mean no. nights per month (tertiles)		1.03 (1.01-1.05)	1.01 (0.99-1.03)		1.02 (0.99-1.04)	1.00 (0.98-1.03)		1.05 (1.03-1.08)	1.03 (1.00-1.06)
Never	308/2187	1 [ref.]	1 [ref.]	222/1373	1 [ref.]	1 [ref.]	86/814	1 [ref.]	1 [ref.]
1-3	228/1500	1.03 (0.88-1.21)	0.99 (0.84-1.17)	187/1214	0.95 (0.80-1.14)	0.93 (0.78-1.12)	41/286	1.35 (0.95-1.91)	1.24 (0.88-1.76)
4-5	199/1255	1.04 (0.88-1.23)	1.02 0.86-1.21)	169/1076	0.96 (0.80-1.16)	0.94 (0.78-1.14)	30/179	1.58 (1.08-2.31)	1.41 (0.97-2.06)
≥6	156/802	1.37 (1.15-1.63)	1.21 (1.01-1.45)	102/511	1.23 (0.99-1.52)	1.11 (0.90-1.38)	54/291	1.75 (1.28-2.40)	1.46 (1.07-2.01)
Missing	37/207			31/147			6/60		
Night worker, period unknown	490/3135			314/1779			176/1356		
P-trend		0.006	0.041		0.017	0.067		0.176	0.317
Cumulative no. nights (tertiles)		1.00 (1.00-1.00)	1.00 (0.99-1.00)		1.00 (0.99-1.00)	1.00 (0.99-1.00)		1.00 (1.00-1.00)	1.00 (1.00-1.00)
Never	308/2187	1 [ref.]	1 [ref.]	222/1373	1 [ref.]	1 [ref.]	86/814	1 [ref.]	1 [ref.]
1-121	180/1207	1.01 (0.85-1.20)	0.98 (0.82-1.16)	148/952	0.96 (0.79-1.16)	0.94 (0.77-1.14)	32/255	1.18 (0.81-1.73)	1.10 (0.75-1.60)
122-240	237/1480	1.07 (0.91-1.25)	1.04 (0.88-1.22)	201/1263	0.98 (0.82-1.17)	0.97 (0.81-1.16)	36/217	1.57 (1.09-2.24)	1.42 (0.99-2.04)
≥241	166/870	1.33 (1.12-1.58)	1.18 (0.99-1.41)	109/586	1.15 (0.93-1.41)	1.05 (0.85-1.29)	57/284	1.90 (1.40-2.57)	1.56 (1.14-2.13)
Missing	37/207			31/147			6/60		
Night worker, period unknown	490/3135			314/1779			176/1356		
P-trend		0.009	0.065		0.113	0.241		0.021	0.109
Mean no. consecutive nights per month (tertiles)		1.05 (1.01-1.08)	1.02 (0.99-1.06)		1.02 (0.98-1.06)	1.00 (0.96-1.04)		1.12 (1.06-1.19)	1.08 (1.02-1.14)
Never	308/2187	1 [ref.]	1 [ref.]	222/1373	1 [ref.]	1 [ref.]	86/814	1 [ref.]	1 [ref.]
1-2	159/1045	1.05 (0.88-1.25)	1.01 (0.84-1.21)	131/829	0.98 (0.80-1.19)	0.97 (0.79-1.18)	28/216	1.22 (0.82-1.82)	1.14 (0.77-1.69)
3	191/1262	1.03 (0.87-1.22)	0.98-0.82-1.16)	150/993	0.93 (0.77-1.13)	0.91 (0.75-1.10)	41/269	1.44 (1.02-2.03)	1.25 (0.88-1.77)
≥4	223/1230	1.21 (1.03-1.43)	1.13 (0.96-1.34)	171/970	1.08 (0.90-1.30)	1.02 (0.85-1.23)	52/260	1.89 (1.38-2.59)	1.60 (1.16-2.19)
Missing	47/227			37/156			10/71		
Night worker, period unknown	490/3135			314/1779			176/1356		
P-trend		0.057	0.161		0.290	0.495		0.039	0.072

Abbreviations: IRR: incidence rate ratio- CI: confidence interval- ref: reference category- no: number. [a]adjusted for age at baseline, [b]adjusted for age, smoking status, menopausal status, alcohol consumption, sports activity, highest education achieved, chronotype, average monthly household income, sleep duration, marital status, number of children and job load at baseline, [c]adjusted for age, smoking status, alcohol consumption, sports activity, highest education achieved, chronotype, average monthly household income, sleep duration, marital status, number of children and job load at baseline

Table S7. Associations of night shift work exposure in 2011 with moderate weight gain (>5%) during 5.5 year follow-up by menopausal status at baseline, based on observed data (n=16 040)

	Premenopausal in 2011			Postmenopausal in 2011		
	n >5% weight gain during follow-up/n total	IRR (95% CI)[a]	IRR (95% CI)[b]	n >5% weight gain during follow-up/n total	IRR (95% CI)[a]	IRR (95% CI)[b]
**Night work exposure in 2011**						
Night work status						
Never	642/2093	1 [ref.]	1 [ref.]	235/1402	1 [ref.]	1 [ref.]
Night work <2011	1257/4201	1.04 (0.96-1.13)	1.05 (0.97-1.13)	663/3540	1.11 (0.97-1.27)	1.11 (0.97-1.27)
Night work in 2011	1205/3707	1.03 (0.95-1.11)	1.00 (0.92-1.08)	257/1097	1.36 (1.15-1.62)	1.27 (1.07-1.51)
Night worker, period unknown	879/2912			473/2423		
Comparison within night workers						
Night work <2011	1257/4201	1 [ref.]	1 [ref.]	663/3540	1 [ref.]	1 [ref.]
Night work in 2011	1205/3707	0.98 (0.92-1.05)	0.95 (0.89-1.02)	257/1097	1.18 (1.01-1.36)	1.10 (0.94-1.27)
Night worker, period unknown	879/2912			473/2423		
Mean no. nights per month (tertiles)		1.01 (0.99-1.03)	1.00 (0.99-1.00)		1.03 (1.01-1.05)	1.02 (0.99-1.04)
Never	642/2093	1 [ref.]	1 [ref.]	235/1402	1 [ref.]	1 [ref.]
1-3	503/1576	1.02 (0.92-1.12)	1.01 (0.92-1.11)	67/317	1.18 (0.93-1.51)	1.12 (0.88-1.43)
4-5	390/1222	0.97 (0.88-1.08)	0.95 (0.86-1.06)	54/225	1.36 (1.05-1.77)	1.26 (0.97-1.64)
≥6	238/688	1.12 (0.99-1.26)	1.03 (0.91-1.16)	110/441	1.45 (1.19-1.77)	1.26 (1.03-1.54)
Missing	74/221			26/114		
Night worker, period unknown	879/2912			473/2423		
*P*trend		0.135	0.500		0.183	0.407
Mean no. consecutive nights per month (tertiles)		1.02 (0.99-1.04)	1.00 (0.99-1.03)		1.06 (1.03-1.11)	1.04 (1.00-1.08)
Never	642/2093	1 [ref.]	1 [ref.]	235/1402	1 [ref.]	1 [ref.]
1-2	362/1152	1.02 (0.92-1.14)	1.01 (0.91-1.13)	61/273	1.26 (0.98-1.62)	1.22 (0.95-1.56)
3	359/1163	0.97 (0.87-1.08)	0.95 (0.85-1.06)	63/317	1.13 (0.88-1.46)	1.03 (0.80-1.32)
≥4	404/1155	1.07 (0.96-1.18)	1.01 (0.92-1.13)	105/386	1.58 (1.29-1.92)	1.37 (1.11-1.67)
Missing	80/237			28/121		
Night worker, period unknown	879/2912			473/2423		
*P*trend		0.241	0.577		0.093	0.231

Abbreviations: IRR: incidence rate ratio- CI: confidence interval- ref: reference category- no: number.

[a]adjusted for age at baseline

[b]adjusted for age, smoking status, alcohol consumption, sports activity, highest education achieved, chronotype, average monthly household income, sleep duration, marital status, number of children and job load at baseline

Table S8. Associations of night shift work exposure in 2011 with development of overweight or obesity during 5.5 year follow-up in a subgroup of nurses with healthy weight at baseline, by menopausal status at baseline, based on observed data (n=9652)

	Premenopausal in 2011			Postmenopausal in 2011		
	n overweight or obesity follow-up/ n healthy weight at baseline	IRR (95% CI)[a]	IRR (95% CI)[b]	n overweight or obesity follow-up/ n healthy weight at baseline	IRR (95% CI)[a]	IRR (95% CI)[b]
**Night work exposure in 2011**						
Night work status						
Never	222/1373	1 [ref.]	1 [ref.]	86/814	1 [ref.]	1 [ref.]
Night work <2011	418/2587	1.00 (0.86-1.16)	1.01 (0.87-1.17)	222/1934	1.09 (0.86-1.37)	1.07 (0.85-1.36)
Night work in 2011	397/2352	1.04 (0.90-1.21)	1.00 (0.86-1.17)	102/592	1.56 (1.16-2.10)	1.41 (1.05-1.90)
Night worker, period unknown	314/1779			176/1356		
Comparison within night workers						
Night work <2011	418/2587	1 [ref.]	1 [ref.]	222/1934	1 [ref.]	1 [ref.]
Night work in 2011	397/2352	1.04 (0.92-1.18)	1.00 (0.87-1.14)	102/592	1.44 (1.11-1.87)	1.30 (0.99-1.70)
Night worker, period unknown	314/1779			176/1356		
Mean no. nights per month (tertiles)		1.02 (0.99-1.04)	1.00 (0.97-1.03)		1.07 (1.04-1.10)	1.04 (1.01-1.08)
Never	222/1373	1 [ref.]	1 [ref.]	86/814	1 [ref.]	1 [ref.]
1-3	159/1017	0.97 (0.80-1.16)	0.95 (0.79-1.15)	25/193	1.22 (0.80-1.85)	1.14 (0.75-1.73)
4-5	129/810	0.98 (0.80-1.20)	0.96 (0.78-1.18)	24/122	1.86 (1.23-2.80)	1.64 (1.09-2.46)
≥6	81/398	1.26 (1.00-1.58)	1.12 (0.89-1.41)	46/221	1.97 (1.42-2.72)	1.60 (1.15-2.23)
Missing	28/127			7/56		
Night worker, period unknown	314/1779			176/1356		
*P*trend		0.030	0.102		0.051	0.147
Mean no. consecutive nights per month (tertiles)		1.02 (0.97-1.06)	1.00 (0.95-1.04)		1.15 (1.08-1.22)	1.09 (1.02-1.17)
Never	222/1373	1 [ref.]	1 [ref.]	86/814	1 [ref.]	1 [ref.]
1-2	122/742	1.02 (0.83-1.24)	1.01 (0.82-1.24)	24/160	1.42 (0.93-2.16)	1.32 (0.87-2.00)
3	112/757	0.91 (0.74-1.13)	0.88 (0.71-1.09)	30/191	1.48 (1.01-2.18)	1.28 (0.87-1.89)
≥4	130/713	1.12 (0.92-1.37)	1.05 (0.86-1.29)	40/181	2.09 (1.49-2.93)	1.71 (1.21-2.41)
Missing	33/140			8/60		
Night worker, period unknown	314/1779			176/1356		
*P*trend		0.335	0.519		0.092	0.264

Abbreviations: IRR: incidence rate ratio- CI: confidence interval- ref: reference category- no: number.
[a]adjusted for age at baseline
[b]adjusted for age, smoking status, alcohol consumption, sports activity, highest education achieved, chronotype, average monthly household income, sleep duration, marital status, number of children and job load at baseline
